# Sex Differentiation of Trabecular Bone Structure Based on Textural Analysis of Pelvic Radiographs

**DOI:** 10.3390/jcm13071904

**Published:** 2024-03-26

**Authors:** Paweł Kamiński, Karolina Nurzynska, Joanna Kwiecień, Rafał Obuchowicz, Adam Piórkowski, Elżbieta Pociask, Aleksandra Stępień, Marcin Kociołek, Michał Strzelecki, Piotr Augustyniak

**Affiliations:** 1 Clinic of Locomotor Disorders, Andrzej Frycz Modrzewski Krakow University, 30-705 Krakow, Poland; pawelkam@mp.pl; 2 Małopolska Orthopedic and Rehabilitation Hospital, Modrzewiowa 22, 30-224 Krakow, Poland; 3 Department of Algorithmics and Software, Silesian University of Technology, 44-100 Gliwice, Poland; 4 Department of Automatic Control and Robotics, AGH University of Krakow, 30-059 Krakow, Poland; kwiecien@agh.edu.pl; 5 Department of Diagnostic Imaging, Jagiellonian University Medical College, Kopernika 19, 31–501 Krakow, Poland; rafalobuchowicz@su.krakow.pl; 6 Department of Biocybernetics and Biomedical Engineering, AGH University of Krakow, 30-059 Krakow, Polandepociask@agh.edu.pl (E.P.); august@agh.edu.pl (P.A.); 7 Independent Researcher, 00-237 Warszawa, Poland; olli.stepien@gmail.com; 8 Institute of Electronics, Lodz University of Technology, 93-590 Lodz, Poland; marcin.kociolek@p.lodz.pl (M.K.); mstrzel@p.lodz.pl (M.S.)

**Keywords:** textural analysis, radiographs, pelvic regions, sex estimation, machine learning

## Abstract

**Objectives:** The purpose of this paper is to assess the determination of male and female sex from trabecular bone structures in the pelvic region. The study involved analyzing digital radiographs for 343 patients and identifying fourteen areas of interest based on their medical significance, with seven regions on each side of the body for symmetry. **Methods:** Textural parameters for each region were obtained using various methods, and a thorough investigation of data normalization was conducted. Feature selection approaches were then evaluated to determine a small set of the most representative features, which were input into several classification machine learning models. **Results:** The findings revealed a sex-dependent correlation in the bone structure observed in X-ray images, with the degree of dependency varying based on the anatomical location. Notably, the femoral neck and ischium regions exhibited distinctive characteristics between sexes. **Conclusions:** This insight is crucial for medical professionals seeking to estimate sex dependencies from such image data. For these four specific areas, the balanced accuracy exceeded 70%. The results demonstrated symmetry, confirming the genuine dependencies in the trabecular bone structures.

## 1. Introduction

There are noticeable disparities between the bone structures of males and females. These differences are attributable to estrogen-induced periosteal growth inhibition, directly proportional to a two-year-earlier maturation in girls than boys [1]. Additionally, hormonal and related structural phenomena resulting from developmental variations in boys and girls lead to different bone geometries, greater bone mass, and increased trabecular thickness in boys [2,3]. Conversely, the inhibitory action of estrogen on osteoclasts throughout adulthood is responsible for bone protection. In postmenopausal women, when estrogen influence is diminished, metabolic changes result in osteoporosis and fragile bones [4]. The differences in trabeculae between men and women are primarily influenced by hormone levels, which are recognized as the most critical factor in sex differences in bone mechanics [5,6]. However, decreased protein intake in elderly individuals with regional differences between men and women was also identified as a possible factor with implications for bone structure diversity [7]. Furthermore, there are differences in the activity bias between men and women, which can impact bone remodeling differently [8].

In our work, we utilized biology-driven variations in bone structure to determine an individual’s sex. Certain bone features, such as trabecular structure, are indistinguishable even to experienced observers like radiologists when examined by the human eye. However, these features can be highlighted through textural analyses [9]. Determining bone morphology, which reflects an individual’s unique features, is crucial in forensic medicine and trauma management. Sex estimation is the initial step in creating an osteological profile, where bones are the only remaining human remains. Differences in sex estimation are influenced, in part, by sex-related variations in pelvic shape due to pubertal dimorphism and different postures and gaits [10]. Subjective and visual methods are commonly used for sex estimation, but they may be inaccurate, and reproducible results are essential [11]. Applying geometric and morphometric methods can achieve high accuracy rates, up to 100%, under conditions where well-defined landmarks are present [12]. However, these techniques are not applicable when only parts of the pelvic bone are available, and other objective bone analyses, such as X-ray analyses, must be performed [13]. Textural analyses can also reflect bone quality, making them helpful in determining the ideal locations for implementing surgical hardware during fracture fixation [14,15,16]. It should be noted that various techniques have been used for age or sex estimation [14,15,16]. In ref. [14], radiograms of different bone structures are utilized to discriminate between specific age ranges of living individuals from the Indian population for legal purposes. For children aged 3 months to 16 years, specific carpal bones and their appearance in anteroposterior (AP) wrist radiographs are associated with particular ages. Additionally, for children over 14 years old, AP radiographs of the pelvis and elbow are performed to discern ages around pre- and early puberty. AP radiographs of the elbow, wrist, or shoulder joint for late puberty are analyzed to enhance age determination. Furthermore, chest radiographs distinguish whether individuals have reached retirement age (60–62). In ref. [15], the sex and age of human patients are determined based on image analysis applied to spine images. In this experiment, CT scans presenting from 4 to 12 vertebrae are employed. Texture-based features and convolutional neural networks are utilized to classify sex, and regression analysis is employed for age estimation ranging from 21 to 84 years. In ref. [16], bone mineral density (BMD) of the cranium and femur fragments is used to estimate the age-at-death and sex of unidentified human remains. The BMD is measured using the dual-energy X-ray absorptiometry technique.

A review of conventional geometric and morphometric approaches utilized on the thoracic vertebrae’s vertebral body and pedicle is provided in ref. [17]. Nowadays, the emerging problem of an ageing population with possible low bone quality and high incidence of pelvic fractures in older women is an essential clinical aspect of study [18,19]. In ref. [20], radiographs of reconstructed cadaver knee joints are utilized to identify the sex of remains from Anglo and African Americans dating back to the late 19th and early 20th centuries. The subjects selected for the experiment have an age of death ranging from 64 to 102 without bone-related diseases. Deep learning algorithms are employed for automatic image analysis.

The aim of our study was to determine the performance of a novel approach to utilizing textural parameters to analyze X-rays of pelvic bones for the determination of bone quality and sex. Additionally, bone regions that best reflect sexual dimorphism were determined. This addresses the need for rapid and effective detection of areas most vulnerable to potential pathological changes.

## 2. Materials and Methods

**Figure 1:**
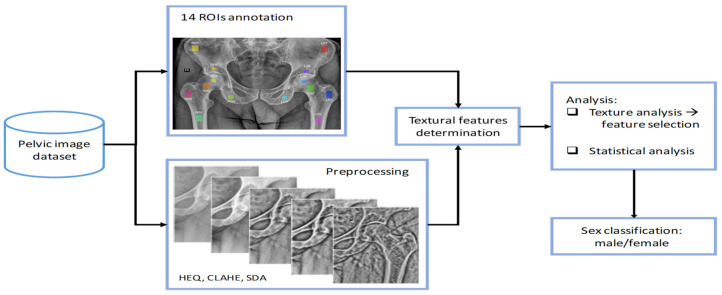
Methodological workflow of the research.

Figure 1 depicts the primary steps of the methodology we present. Our dataset comprises 343 anonymized images of pelvis digital radiography (DR) scans. Initially, these data were recorded in 16-bit DICOM (Digital Imaging and Communication in Medicine) format, necessitating preprocessing to accommodate the 8-bit data supported by image analysis tools. Subsequently, we identified fourteen distinct regions of interest (ROIs) in each pelvis, seven on each side of the body. For each ROI, we calculated a set of textural features using the pyRadiomics [21] library accessible in Python. Subsequently, we determined the correlation of these features with patient sex, and because there is no simple feature that would state the patient sex with a high probability, more complex statistical models were prepared. Given the larger number of textural features than the number of samples we could use to train the model, we applied feature selection methods to determine the most promising features, which were then utilized to train the classification model. Separate models were prepared for each ROI in the pelvis.

### 2.1. Dataset Description

The dataset includes 343 radiographic images of the pelvis selected from a larger dataset of 684 images. This selection ensures that any severe medical conditions, low-quality images, or presence of prostheses did not influence the data. The radiographic images were acquired from Caucasian individuals, including 253 women and 90 men, routinely examined during hospital admission. The age range of the women was between 34 and 94, while the men’s age range was between 26 and 90, with an average age of 65.4 ± 12.3 and 60 ± 13.7 for women and men, respectively. The images were taken using the Visaris Avanse DR (Visaris, Serbia), a digital radiography imaging system, and stored as 12-bit (16-bit allocated) DICOM data with a pixel spacing of 0.13256 mm × 0.13256 mm. All images were anonymized, and the patient’s sex was saved in the metadata associated with the dataset. The local Institutional Ethical Board approved this retrospective study, as patient involvement was omitted; therefore, additional consents or declarations were not required (A.I.060.3.2024).

### 2.2. ROI Annotation

**Figure 2:**
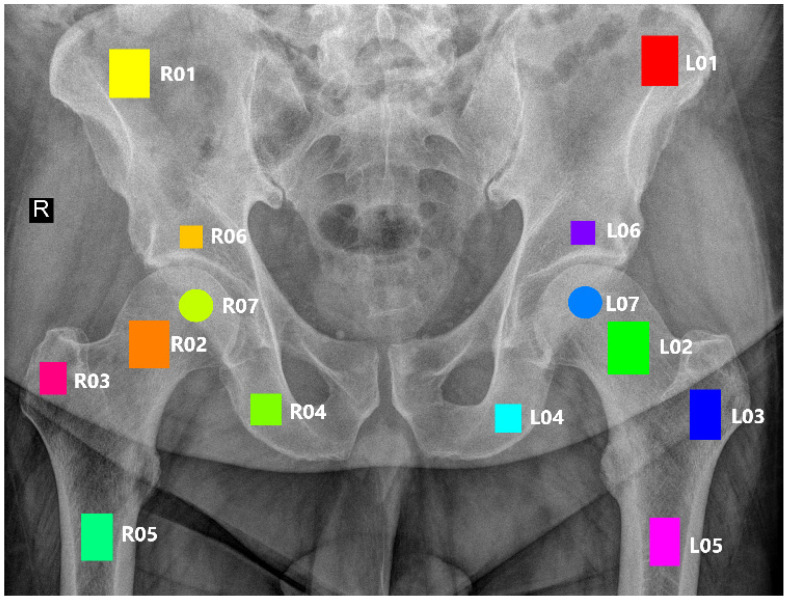
Marked regions of interest.

By employing the qMaZda 19.02 software [22], twelve rectangular and two circular regions of interest (ROIs) were meticulously annotated onto X-ray images of the pelvic bones. These ROIs were designated by experienced radiologists with at least six years of specialization in musculoskeletal structures. In order to ensure accurate placement of the ROIs, images without prostheses were prioritized, as these devices can obstruct the site of interest. The spatial arrangement of the ROIs in relation to the anatomical structures is illustrated in Figure 2 and described in detail in Table 1. The ROIs are distinguished by unique colors for easy identification.

### 2.3. Preprocessing

**Figure 3:**
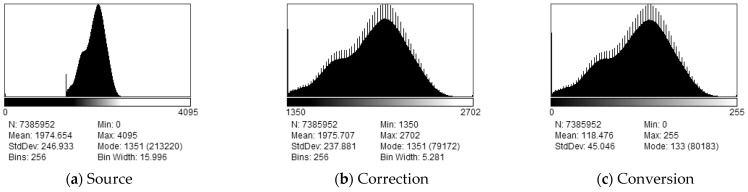
Rescaling to 8-bit data: (**a**) histogram of the source DICOM file; (**b**) histogram after patient side marker correction; (**c**) histogram after conversion to 8 bit.

**Figure 4:**
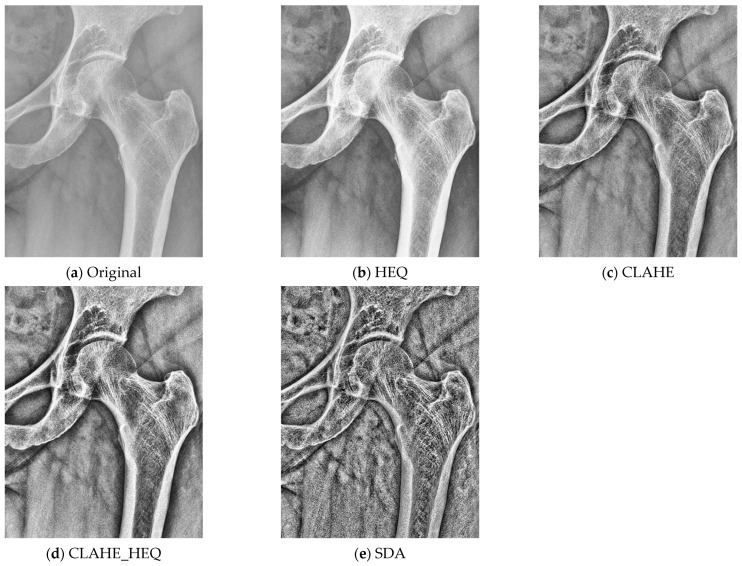
Comparison between the original and preprocessed images for enhancing the trabecular structure of the bones: (**a**) original image; (**b**) image after HEQ; (**c**) image after CLAHE; (**d**) image after combination of CLAHE and HEQ; (**e**) image after SDA.

The research methodology entailed the following procedures aimed at achieving optimal texture parameters. The images, initially in DICOM 16-bit format, were encoded with only 12 bits of data (0–4095). To normalize the images, we proportionally scaled them to 8 bits, using each image’s maximum and minimum pixel brightness to delineate the valid information range. In this process, we disregarded the side indicator labeled ‘R’ as it includes the full range of values. Figure 3 illustrates this procedure through a graphical representation of the histogram distribution of pixel brightness values before and after scaling. Subsequently, the original image was enhanced with additional filters to emphasize the visual changes in the trabecular structure of the bones. This step involved applying histogram equalization (HEQ), contrast-limited adaptive histogram equalization (CLAHE) [23], their combination (CLAHE_HEQ), and the statistical dominance algorithm (SDA) [24]. For CLAHE, we used the default values in the ImageJ/Fiji package [25], including a block size of 127 pixels, 256 histogram bins, and a maximum slope of 3. For SDA, we assumed a radius of 50 pixels. Figure 4 demonstrates the impact of the preprocessing methods.

### 2.4. Textural Features

- After designating all the regions of interest (ROIs), we proceeded with the determination of textural features based on pyRadiomics [21]. All available textural parameters were computed. In the following text, we will limit ourselves to a concise list of the primary methods employed in our research. The set of parameters was derived from various statistical image descriptors and comprised the following groups (as described in refs. [21,26]):
- First-order features (FOFs) are based on simple statistical information that can be determined from an image histogram. These features include the 10th percentile, 90th percentile, energy, total energy, entropy, minimum, maximum, mean, median, range, inter-quartile range, mean absolute deviation, robust mean absolute deviation, root mean squared, kurtosis, skewness, standard deviation, uniformity, and variance.
- The gray-level co-occurrence matrix (GLCM) derives textural information by considering the spatial distribution of pixel brightness in the image. It consists of the following features: autocorrelation, joint average, cluster prominence, cluster shade, cluster tendance, contrast, correlation, difference average, difference entropy, difference variance, joint energy, joint entropy, informational measure of correlation (IMC) 1, informational measure of correlation (IMC) 2, inverse difference moment, maximal correlation coefficient, inverse difference moment normalized, inverse difference, inverse difference normalized, inverse variance, maximum probability, sum entropy, and the sum of squares.
- The grey-level size zone matrix (GLSZM) provides a statistical representation by estimating a bivariate conditional probability density function of the image distribution values. This matrix takes into account various aspects such as minor area emphasis, significant area emphasis, grey-level non-uniformity, size-zone non-uniformity, zone percentage, grey-level variance, zone variance, zone entropy, low grey-level zone emphasis, high grey-level zone emphasis, small-area low grey-level emphasis, small-area high grey-level emphasis, large-area low grey-level emphasis, and large-area high grey-level emphasis.
- The grey-level run length matrix (GLRLM) considers that images with more frequent changes in pixel brightness next to each other have better contrast, while those where one color is kept longer are blurred. Therefore, the length of pixels with the same color in a linear neighborhood is considered as basic information and used to calculate the following features: short-run emphasis, long-run emphasis, grey-level non-uniformity, grey-level non-uniformity normalized, run length non-uniformity, run length non-uniformity normalized, run percentage, grey-level variance, run variance, run entropy, low grey-level run emphasis, high grey-level run emphasis, short-run low grey-level emphasis, short-run high grey-level emphasis, long-run low grey-level emphasis, and long-run high-grey level emphasis.
- The gray-level dependence matrix (GLDM) evaluates the relationships between the brightness of neighboring pixels in a rectangular area. The following features are derived from this analysis: small dependence emphasis, large dependence emphasis, gray-level non-uniformity, dependence non-uniformity, dependence non-uniformity normalized, gray-level variance, dependence variance, dependence entropy, low gray-level emphasis, high gray-level emphasis, small dependence low gray-level emphasis, small dependence high-gray level emphasis, large dependence low gray-level emphasis, and large dependence high gray-level emphasis.
- The neighboring grey tone difference matrix (NGTDM) determines image information by analyzing the rectangular region and comparing its central pixel brightness with the average one for the whole area. It calculates coarseness, contrast, business, complexity, and strength.
- The gradient map features derive information from the changing magnitude within an ROI. This method calculates five parameters: mean, variance, skewness, kurtosis, and nonzeros.
- The first-order autoregressive model assumes the dependence of pixel intensities of adjacent pixels and aims to find weights of gradient directions that best correlate to the region’s pixel distributions. In consequence, four parameters are calculated.
- The Haar wavelet transform decomposes the image into several sub bands of energies and calculates their frequency characteristics. There are four scale transformations, and the features describe spatial combinations of the resulting low-pass and high-pass values.
- The Gabor transform decomposes the image into frequency components. The valid frequencies are found by convolving the image with proper complex number kernels, while stored parameters describe the Gaussian envelope’s frequency, orientation, and standard deviation.
- The histogram of oriented gradients determines the features from histograms representing the frequency of orientations found in the image. The feature vector constitutes a normalized histogram.

Based on our previous research, we understand that data normalization is essential for determining accurate textural features. Therefore, we utilized the original pixel brightness distribution for textural feature calculation (D) and employed additional normalization techniques already present in the qMaZda software. Specifically, we implemented mean value and standard deviation normalization (S), min–max image normalization (M), and the removal of the first and last percentiles of the region histogram (N). Given that the ROIs are relatively small, using the full range of possible pixel brightness values (0–255) can lead to statistically unstable structures in feature calculations. To address this issue, we enabled the calculation of features for images with quantized pixel values, following the qMaZda approach. It is proved that a reduction in image depth increases the texture utility [27,28,29].

The number of features calculated for each ROI is significantly larger than the number of samples. This excess of data makes training a classification model difficult due to data redundancy and override of the significant structures with less critical but numerous information. Therefore, we decided to apply feature selection methods in order to diminish the number of features used to train the model and choose the most characteristic ones. In the experiments, several methods for feature vector reduction were evaluated: four correlations (Fisher, Spearman, Pearson, Kendall), mutual information maximization (MIM), max-relevance and min-redundancy (MRMR), mutual joint information (JMI), conditional information feature extraction (CIFE), and principal component analysis (PCA). We also compared several classifiers: random forest (RF), logistic regression (LR), and support vector machines (SVM) with linear and radial basis functions (RBFs) for the kernel.

## 3. Results

We conducted numerous independent experiments using different input data normalization techniques to determine which method better visualizes the trabecular structure: HEQ, CLAHE, CLAHE + HEQ, SDA, or the original data. Normalization improves data quality and reduces noise, which could increase the variation in texture feature values. This is particularly relevant for methods that enhance local contrast (CLAHE) or normalize the local context (SDA). Following this, each ROI was normalized using the D, N, or S approaches, and the number of bits for image quantization was chosen (5–8). A separate model was trained for each potential combination of these approaches. Additionally, we evaluated all possible combinations of feature selection methods and classification approaches mentioned earlier. This resulted in thousands of outcomes, providing valuable insight into the complexity of sex classification based on the texture feature extraction of the source DICOM file.

To ensure the stability of the results and prevent dependence on sample splitting between training and validation, we applied the five-fold cross-validation method in each experiment. The results reported in this study are the average of the five runs. Next, to address the issue of unbalanced data, we selected the balanced accuracy (BACC) metric to compare outcomes. BACC is directly proportional to sensitivity and specificity (1), the two most important factors defining a good classifier. Table 2 gathers the highest BACC scores recorded for each ROI, considering several bits used for feature calculation. This table presents the maximum and average BACC achieved for one region.

(1) $$BACC=\frac{\mathrm{Sensitivity}+\mathrm{Specificity}}{2},$$

**Figure 5:**
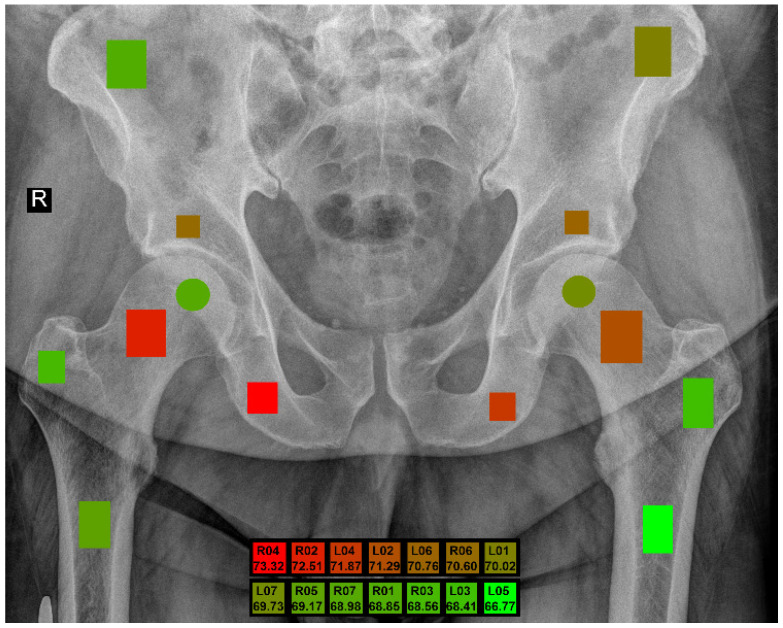
Marked regions of interest with the best BACC.

The best outcomes, with over 70% balanced accuracy (BACC) of sex distinction, were achieved for four regions: L02, R02, L04, and R04, regardless of the number of bits used for feature calculation. Figure 5 visually presents the highest BACC score recorded for each region of interest (ROI) from all evaluated experiments. The research demonstrates that sex can be determined with 73.32% balanced accuracy obtained for the ischium on the right side (R04).

Moreover, we examined whether providing the classifier with information from various preprocessing and feature extraction methods influenced the classifier’s quality. Table 3 compiles the top 10 BACC results for the L02, R02, L04, and R04 regions, along with the number of bits used for feature calculation. The table details the methods of normalization (column “Preproc”), feature selection (column “Filter”), and classification (column “Classifier”) used to build the model. This table provides comprehensive information on the statistical results of our experiments, including other metrics such as accuracy, sensitivity (recall), specificity, and F1-score. However, due to the high dataset imbalance, balanced accuracy is the most optimal metric for comparison. From the results inspection, it is difficult to pinpoint the best set of methods for sex classification and clearly state which classifier or feature selection method is the best. Nonetheless, the similarity in values and repeatability confirms the reliability of the obtained results. We also conducted another study where a smaller number of features were selected for the feature vector, and we found that using only six features selected by one of the following methods: multi-CIFE, multi-JMI, multi-MIM, PCA, uni-Kendall, and uni-Pearson did not result in a significant loss in the quality of the results.

## 4. Discussion

The process of bone analysis has a range of applications in legal contexts, where it is used to determine human origin or personal data in fields like forensic medicine and physical anthropology. In this procedure, the pelvis is considered the best bone for sex determination, with metric and morphological methods achieving reported accuracies of 92% and 91%, respectively. In fact, the pelvis outperforms other bones in this regard [30]. Morphometric methods that involve the use of coordinates have been shown to achieve up to 100% accuracy in sex prediction, but these techniques can be challenging when the coordinates are not always identifiable [31]. As Fukuta suggests [32], new approaches to bone analysis have achieved up to 90% accuracy in sex estimation. In recent studies, convolutional neural networks have been used to evaluate three-dimensional reconstructed tomography images of pelvic bones, with transfer learning applied to achieve these goals. The final result of a correct assignment rate higher than 90% was obtained. Additionally, the reconstruction and analysis of the cortex has been proposed as a method for determining cancellous bone quality [33].

Our research has shown that the methods we discussed above yield results that are comparable to our own findings in context of sex determination from bone structures. An additional benefit of our work is that we identified 14 regions of interest (ROIs) that accurately discriminate between the sexes. Out of these fourteen ROIs, four were found to be particularly effective. These ROIs were selected experimentally based on their ability to reflect bone morphology-driven dimorphism, and they were located in various parts of the pelvic bones and hips. Our approach, as well as the other solutions mentioned in this paper, relies on selected regions of interest to determine sex based on textural features. The best distinction between sex was noticed in the regions of femoral neck and ischium. By identifying these regions, we can ensure that we are analyzing the parts of the pelvic bones that provide the most accurate results. Our research suggests that the central part of the femoral head, where the principal tensile trabeculae arc is located, is one of the most effective areas for determining sex [34]. Additionally, we found that the load-bearing area of the hip acetabulum was also a suitable location for analyzing sex differences. These findings are consistent with previous studies that determined sex primarily from the most loaded parts of the skeletal structures in the hip [35]. The importance of these ROIs is at least partially related to hormonal influences on compact bone and the increased thickness of trabeculae in men [5,36]. The observations made in this work are also applicable in forensic medicine to determine the sex of bone remnants. Our research has implications for medical applications, as the proposed methods for determining trabecular structure and bone quality markers can also be used to identify the parts of the pelvis where surgical fixation after complex injuries can be securely implemented. Determining the sites with the highest possible bone strength and capacity to absorb screws is crucial for the postsurgical period [37].

While evaluating various methodologies, it was intriguing to observe that limiting the number of textural features to ten elements (reduced from hundreds) was sufficient to describe the characteristic differences between the sexes. Apart from the reported feature vector with a length of ten, we also experimented with a shorter description of the imagery data, which yielded slightly inferior results. However, the lack of repetitiveness between the results mostly concerned us as it suggested overtrained models. In the case of models prepared with ten features, this issue did not arise. We also investigated whether different approaches to feature selection influenced the outcomes, but no such correlation was noticed. Another aspect of this research involved selecting the optimal classification methodology for the sex determination task from the region of interest marked in the pelvis. This aspect of the experiments showed that all classification models have similar discriminative capabilities. Thus, it is challenging to determine the best one. Next, the research addressed the problem of bit reduction in the image, which might be necessary to quantize the data, as the textural features should create more stable statistical structures. However, a strong correlation was not observed here. Nonetheless, we confirmed that applying a smaller number of bits is sufficient for bone texture description, as evident in Table 3, where many of the best scores are achieved for fewer bits. Finally, we evaluated the influence of data preprocessing with filters on the final textural feature calculation. However, as shown in Table 3, the normalization approach should be applied, as most outcomes for normalized data exist. However, again, it is not possible to determine which approach is the best in this case.

Textural assessment of bone structural characteristics is a straightforward, efficient, and noninvasive method for estimating bone structure. This X-ray-based technique’s effectiveness can be compared to mechanical testing, X-ray analyses, CT, DeXa, and MR. Willet [38] proposed bone collagen structure estimation using thermomechanical methods to test bones associated with the final decomposition of the specimen. Another analytical approach was proposed by Saito [39], where bone mineralization was enzymatically tested, and final bone derangement was also performed. Mechanical testing was proposed by Ebraheim [40], who effectively assessed the sites of the most vital parts of the sacrum. The techniques mentioned above are precise, with high specificity reaching over 90% in chosen methods; however, they are linked to complete derangement of bone. The magnetic resonance technique relates to estimating water content in the tissue; however, it is costly and requires sophisticated equipment. The method proposed by Nyman [41] presented that the ratio of water-bound molecules to free water in cancellous bone can be correlated with the sex and age of the specimen. High-resolution peripheral quantitative computed tomography (HR-pQCT) as a modality implemented for mineral density assessment was used in the femur by Kirhoff [42]. The technique was influential in estimating the specimen’s sex and bone quality at the expense of a higher radiation dose than an X-ray. Micro CT was utilized by Greenwood [43] to effectively correlate parameters related to sex and bone quality with microarchitecture evaluated with low-dose computed tomography. The X-ray-based techniques proposed in our paper offer a low radiation dose compared to CT, low cost compared to MR, and simplicity compared to mechanical testing, making them user and patient-friendly.

A limitation of our study is the unified cohort of Caucasian (European) origin patients from one geographical region and the limited number of participants. There needs to be further comparison of different methods. 

## 5. Conclusions

Our team conducted a comprehensive analysis of various preprocessing techniques and developed a novel method for bone assessment that extracts biological information. This innovative approach integrates non-parametric methods and textural analyses, allowing for precise determination of patient sex. Our technique’s effectiveness in recognizing and quantifying hormonal status, influenced by subtle changes in bone trabecular structure, was evident. The high sensitivity of our approach makes it applicable in both clinical and forensic studies. In future investigations, we plan to compare our current results with densitometry methods and expand our study to include a larger patient population.

## Figures and Tables

**Table 1:** Depicted ROIs with corresponding anatomical structure.

ROI Left	ROI Right	Anatomical Structure
L01	R01	Wing of ilium
L02	R02	Neck of femur
L03	R03	Greater trochanter
L04	R04	Ischium
L05	R05	Shaft of femur
L06	R06	Hip bone above the acetabulum
L07	R07	Femur head (center)

**Table 2:** Results of BACC (in [%]).

ROI	5 Bit	6 Bit	7 Bit	8 Bit	Max	Avg
L01	66.23	68.86	69.19	70.02	70.02	68.58
L02	71.29	70.46	70.03	70.26	71.29	70.51
L03	68.10	68.41	67.41	68.01	68.41	67.98
L04	71.87	71.46	71.21	71.37	71.87	71.48
L05	64.99	66.77	63.63	63.63	66.77	64.76
L06	70.76	68.31	68.72	69.11	70.76	69.23
L07	69.65	69.59	69.73	69.16	69.73	69.53
R01	67.04	68.85	67.34	66.81	68.85	67.51
R02	71.78	71.59	72.51	71.30	72.51	71.80
R03	68.56	66.46	67.47	68.38	68.56	67.72
R04	73.32	70.48	72.64	71.30	73.32	71.94
R05	69.17	67.42	67.57	68.98	69.17	68.29
R06	67.92	68.78	70.60	69.11	70.60	69.10
R07	66.70	67.40	68.98	67.27	68.98	67.59

**Table 3:** Results obtained for most important ROIs (in [%]).

ROI	Bit	Preproc	Filter	Classifier	Accuracy	BACC	Sensitivity	Specificity	F1-Score
L02	5	CLAHE	multi-MIM	SVM_linear	68.83	71.29	75.11	67.47	56.63
	6	CLAHE	multi-MIM	SVM_rbf	66.23	70.46	77.63	63.28	54.71
	6	CLAHE	multi-MIM	SVM_rbf_p	66.23	70.46	77.63	63.28	54.71
	5	SDAr50	multi-CIFE	RF_d4_l5	72.05	70.36	64.87	75.85	54.62
	8	CLAHE	multi-JMI	SVM_rbf	67.70	70.26	74.50	66.02	54.96
	8	CLAHE	multi-JMI	SVM_rbf_p	67.70	70.26	74.50	66.02	54.96
	5	CLAHE+HEQ	uni-Kendall	LR_d	68.85	70.13	70.52	69.74	54.53
	6	SDAr50	multi-CIFE	SVM_rbf	68.24	70.06	72.89	67.23	54.19
	6	SDAr50	multi-CIFE	SVM_rbf_p	68.24	70.06	72.89	67.23	54.19
	7	CLAHE	multi-JMI	SVM_rbf	66.81	70.03	75.67	64.40	54.68
L04	5	none	uni-Pearson	RF_d4_l5	72.04	71.87	68.96	74.79	56.61
	5	none	uni-Pearson	SVM_rbf	71.75	71.74	69.06	74.43	56.28
	5	none	uni-Pearson	SVM_rbf_p	71.75	71.74	69.06	74.43	56.28
	6	HEQ	multi-MIM	SVM_linear	68.53	71.46	75.33	67.59	55.76
	6	HEQ	uni-Fisher	LR_d	70.29	71.45	71.32	71.58	55.07
	5	HEQ	PCA	SVM_linear	69.99	71.37	72.07	70.67	55.84
	8	CLAHE+HEQ	PCA	LR_d	70.55	71.37	71.10	71.64	54.79
	8	CLAHE+HEQ	PCA	SVM_linear	70.55	71.37	71.10	71.64	54.79
	5	HEQ	PCA	LR_d	69.40	71.30	73.12	69.47	55.71
	6	HEQ	uni-Fisher	SVM_linear	70.00	71.26	71.32	71.19	54.77
R02	7	SDAr50	multi-JMI	SVM_rbf	67.43	72.51	80.41	64.60	58.26
	7	SDAr50	multi-JMI	SVM_rbf_p	67.43	72.51	80.41	64.60	58.26
	7	SDAr50	multi-JMI	LR_d	69.16	72.42	76.92	67.93	57.46
	5	SDAr50	multi-CIFE	LR_d	70.00	71.78	73.59	69.97	56.40
	5	SDAr50	PCA	SVM_rbf	71.75	71.67	68.96	74.38	56.16
	5	SDAr50	PCA	SVM_rbf_p	71.75	71.67	68.96	74.38	56.16
	7	SDAr50	PCA	LR_d	67.95	71.62	77.23	66.01	55.71
	6	CLAHE+HEQ	PCA	LR_d	70.02	71.59	72.16	71.02	55.85
	7	SDAr50	PCA	SVM_linear	67.66	71.45	77.23	65.66	55.57
	6	CLAHE+HEQ	PCA	SVM_rbf	70.89	71.44	69.95	72.93	55.95
R04	5	CLAHE+HEQ	multi-CIFE	SVM_rbf	70.58	73.32	77.31	69.33	57.94
	5	CLAHE+HEQ	multi-CIFE	SVM_rbf_p	70.58	73.32	77.31	69.33	57.94
	7	CLAHE+HEQ	multi-CIFE	SVM_rbf	69.12	72.64	78.25	67.03	57.03
	7	CLAHE+HEQ	multi-CIFE	SVM_rbf_p	69.12	72.64	78.25	67.03	57.03
	5	CLAHE+HEQ	multi-CIFE	RF_d4_l5	72.33	71.41	67.48	75.33	56.30
	8	CLAHE+HEQ	multi-CIFE	SVM_rbf	67.38	71.30	77.29	65.31	55.93
	8	CLAHE+HEQ	multi-CIFE	SVM_rbf_p	67.38	71.30	77.29	65.31	55.93
	5	none	uni-Pearson	SVM_rbf	68.88	70.66	72.22	69.11	56.22
	5	none	uni-Pearson	SVM_rbf_p	68.88	70.66	72.22	69.11	56.22
	6	CLAHE+HEQ	multi-CIFE	SVM_rbf	66.81	70.48	76.08	64.88	54.85

## Data Availability

Data can be made available upon request by contacting the corresponding author.

## References

[1] Nieves J.W., Formica C., Ruffing J., Zion M., Garrett P., Lindsay R., Cosman F. (2004). Males Have Larger Skeletal Size and Bone Mass than Females, Despite Comparable Body Size. J. Bone Miner. Res..

[2] Seeman E. (2003). The structural and biochemical basis of the gain and loss of bone strength in women and men. Endocrinol. Metab. Clin. N. Am..

[3] Seeman E. (2001). Sexual dimorphism in skeletal size, density and strength. J. Clin. Endocrinol. Metab..

[4] Khosla S., Oursler M.J., Monroe D.G. (2012). Estrogen and the Skeleton. Trends Endocrinol. Metab..

[5] Noirrit-Esclassan E., Valera M.-C., Tremollieres F., Arnal J.-F., Lenfant F., Fontaine C., Vinel A. (2021). Critical Role of Estrogens on Bone Homeostasis in Both Male and Female: From Physiology to Medical Implications. Int. J. Mol. Sci..

[6] Demontiero O., Vidal C., Duque G. (2011). Aging and bone loss: New insights for the clinician. Ther. Adv. Musculoskelet. Dis..

[7] Hannan M.T., Tucker K.L., Dawson-Hughes B., Cupples L.A., Felson D.T., Kiel D.P. (2000). Effect of Dietary Protein on Bone Loss in Elderly Men and Women: The Framingham Osteoporosis Study. J. Bone Miner. Res..

[8] Christen P., Ito K., Ellouz R., Boutroy S., Sornay-Rendu E., Chapurlat R.D., van Rietbergen B. (2014). Bone remodelling in humans is load-driven but not lazy. Nat. Commun..

[9] Kamiński P., Obuchowicz R., Stępień A., Lasek J., Pociask E., Piórkowski A. (2023). Correlation of Bone Textural Parameters with Age in the Context of Orthopedic X-ray Studies. Appl. Sci..

[10] Lewis C.L., Laudicina N.M., Khuu A., Loverro K.L. (2017). The Human Pelvis: Variation in Structure and Function During Gait. Anat. Rec..

[11] Rajasekhar S., Vasudha T.K., Aravindhan K. (2017). Sex Determination by Biometry of Anterior Features of Human Hip Bones in South Indian Population. J. Clin. Diagn. Res..

[12] Spradley M.K. (2016). Metric Methods for the Biological Profile in Forensic Anthropology: Sex, Ancestry, and Stature. Acad. Forensic Pathol..

[13] de Boer H.H., Roberts J., Delabarde T., Mundorff A.Z., Blau S. (2020). Disaster victim identification operations with fragmented, burnt, or commingled remains: Experience-based recommendations. Forensic Sci. Res..

[14] Bhardwaj V., Kumar I., Aggarwal P., Singh P.K., Shukla R., Verma A. (2024). Demystifying the Radiography of Age Estimation in Criminal Jurisprudence: A Pictorial Review. Indian J. Radiol. Imaging.

[15] Nurzynska K., Piórkowski A., Strzelecki M., Kociołek M., Banyś R.P., Obuchowicz R. (2024). Differentiating age and sex in vertebral body CT scans—Texture analysis versus deep learning approach. Biocybern. Biomed. Eng..

[16] Paschall A., Ross A.H. (2018). Biological sex variation in bone mineral density in the cranium and femur. Sci. Justice.

[17] Sakaran R., Alias A., Woon C.K., Ku Mohd Noor K.M., Zaidun N.H., Zulkiflee N.D.I., Lin N.W., Chung E. (2023). Sex estimation on thoracic vertebrae: A systematic review. Transl. Res. Anat..

[18] Romagnoli E., Carnevale V., Nofroni I., D’Erasmo E., Paglia F., De Geronimo S., Pepe J., Raejntroph N., Maranghi M., Minisola S. (2004). Quality of life in ambulatory postmenopausal women: The impact of reduced bone mineral density and subclinical vertebral fractures. Osteoporos Int..

[19] Buller L.T., Best M.J., Quinnan S.M. (2016). A nationwide analysis of pelvic ring fractures: Incidence and trends in treatment, length of stay, and mortality. Geriatr. Orthop. Surg. Rehabil..

[20] Oura P., Junno J.A., Hunt D., Lehenkari P., Tuukkanen J., Maijanen H. (2023). Deep learning in sex estimation from knee radiographs—A proof-of-concept study utilizing the Terry Anatomical Collection. Leg. Med..

[21] van Griethuysen J.J.M., Fedorov A., Parmar C., Hosny A., Aucoin N., Narayan V., Beets-Tan R.G.H., Fillion-Robin J.-C., Pieper S., Aerts H.J.W.L. (2017). Computational Radiomics System to Decode the Radiographic Phenotype. Cancer Res..

[22] Szczypiński P.M., Strzelecki M., Materka A., Klepaczko A., Kącki E., Rudnicki M., Stempczyńska J. (2009). MaZda—The Software Package for Textural Analysis of Bio-medical Images. Computers in Medical Activity.

[23] Pizer S., Amburn E., Austin J., Cromartie R. (1987). Adaptive histogram equalization and its variations. Comput. Vis. Graph. Image Process..

[24] Piorkowski A. (2016). A Statistical Dominance Algorithm for Edge Detection and Segmentation of Medical Images. Proceedings of the Information Technologies in Medicine: 5th International Conference, ITIB 2016, Kamień Śląski, Poland, 20–22 June 2016.

[25] Schneider C.A., Rasband W.S., Eliceiri K.W. (2012). NIH Image to ImageJ: 25 years of image analysis. Nat. Methods.

[26] Obuchowicz R., Nurzynska K., Pierzchała M., Piorkowski A., Strzelecki M. (2023). Texture analysis for the bone age assessment from MRI images of adolescent wrists in boys. J. Clin. Med..

[27] Kociołek M., Strzelecki M., Obuchowicz R. (2020). Does image normalization and intensity resolution impact texture classification?. Comput. Med. Imaging Graph..

[28] Mazur P., Choraś M., Choraś R.S., Kurzyński M., Trajdos P., Pejaś J., Hyla T. (2022). The Influence of Bit-Depth Reduction on Correlation of Texture Features with a Patient’s Age. Progress in Image Processing, Pattern Recognition and Communication Systems.

[29] Strzelecki M., Kociołek M., Materka A. (2016). On the Influence of Image Features Wordlength Reduction on Texture Classification. Proceedings of the International Conference on Information Technologies in Biomedicine, ITIB 2018, Kamień Śląski, Poland, 18–20 June 2018.

[30] Bruzek J., Murail P. (2006). Methodology and reliability of sex determination from the skeleton. Forensic Anthropology and Medicine.

[31] Bytheway J.A., Ross A.H. (2010). A geometric morphometric approach to sex determination of the human adult os coxa. J. Forensic Sci..

[32] Fukuta M., Kato C., Biwasaka H., Usui A., Horita T., Kanno S., Kato H., Aoki Y. (2020). Sex estimation of the pelvis by deep learning of two-dimensional depth images generated from homologous models of three-dimensional computed tomography images. Forensic Sci. Int. Rep..

[33] Poole K.E.S., Treece G.M., Mayhew P.M., Vaculík J., Dungl P., Horák M., Štěpán J.J., Gee A.H. (2012). Cortical Thickness Mapping to Identify Focal Osteoporosis in Patients with Hip Fracture. PLoS ONE.

[34] Lu Y., Wang L., Hao Y., Wang Z., Wang M., Ge S. (2013). Analysis of trabecular distribution of the proximal femur in patients with fragility fractures. BMC Musculoskelet. Disord..

[35] Purkait R. (2003). Sex determination from femoral head measurements: A new approach. Leg. Med..

[36] DiGirolamo D.J., Clemens T.L., Kousteni S. (2012). The Skeleton as an Endocrine Organ. Nat. Rev. Rheumatol..

[37] Telfer S., Brunnquell C.L., Allen J.D., Linnau K.F., Zamora D., Kleweno C.P. (2021). The Effect of Age and Sex on Pelvic Bone Density Measured Opportunistically in Clinical CT Scans. J. Orthop. Res..

[38] Willett T.L., Dapaah D.Y., Uppuganti S., Granke M., Nyman J.S. (2019). Bone Collagen Network Integrity and Transverse Fracture Toughness of Human Cortical Bone. Bone.

[39] Saito M., Fujii K., Soshi S., Tanaka T. (2006). Reductions in Degree of Mineralization and Enzymatic Collagen Cross-Links and Increases in Glycation-Induced Pentosidine in the Femoral Neck Cortex in Cases of Femoral Neck Fracture. Osteoporos. Int..

[40] Ebraheim N., Sabry F.F., Nadim Y., Xu R., Yeasting R.A. (2000). Internal Architecture of the Sacrum in the Elderly. Spine.

[41] Ni Q., Nyman J.S., Wang X., Santos A.D.L., Nicolella D.P. (2007). Assessment of Water Distribution Changes in Human Cortical Bone by Nuclear Magnetic Resonance. Meas. Sci. Technol..

[42] Kirchhoff C., Braunstein V., Milz S., Sprecher C.M., Kirchhoff S., Graw M., Imhoff A.B., Hinterwimmer S. (2012). Age and Gender as Determinants of the Bone Quality of the Greater Tuberosity: A HR-PQCT Cadaver Study. BMC Musculoskelet. Disord..

[43] Greenwood C., Clement J., Dicken A., Evans P., Lyburn I., Martin R.M., Stone N., Zioupos P., Rogers K. (2018). Age-Related Changes in Femoral Head Trabecular Microarchitecture. Aging Dis..

